# Evaluation of a new system to determine wireless real-time skin temperature

**DOI:** 10.1007/s10973-026-15570-9

**Published:** 2026-05-11

**Authors:** Hein A. M. Daanen, Nienke J. Haakma, Hashim A. Quraishi, Lisa Klous, Wouter D. van Marken Lichtenbelt, Coen C. W. G. Bongers

**Affiliations:** 1 Department of Human Movement Sciences, Faculty of Behavioural and Movement Sciences, Amsterdam Movement Sciences, Vrije Universiteit Amsterdam, Van der Boechorststraat 7, 1081BT Amsterdam, The Netherlands; 2 TNO Defence, Safety and Security, Soesterberg, The Netherlands; 3 Department of Nutrition and Movement Sciences, School of Nutrition and Translational Research in Metabolism (NUTRIM), Maastricht University, Maastricht, The Netherlands; 4 School of Sport and Exercise, HAN University of Applied Sciences, Nijmegen, The Netherlands; 5 Department of Medical Biosciences, Exercise Physiology Research Group, Radboud University Medical Center, Nijmegen, The Netherlands

**Keywords:** Temperature, Skin, Validity, Reliability, Response time

## Abstract

To evaluate the body’s thermal status, it is essential to determine the mean skin temperature, ideally using wireless technology with real-time feedback. Such a system was not yet available. Therefore, the aim of current study was to examine the in- and ex-vivo validity and reliability of an innovative skin temperature sensor. New skin temperature sensors, named eTemp Performance, were developed with memory and direct read-out capabilities as an alternative to commonly used wireless iButton sensors that provide no real-time feedback. The time constant and precision of the sensors were evaluated by immersion in water baths and human experiments during exercise in the heat. The absolute skin temperature values measured with the eTemp Performance sensors and iButtons were compared to a calibrated gold standard with a variation during measurements of 0.12 °C. The time constant in water of the eTemp Performance sensors was 10.5 ± 1.1 s and 25.8 ± 3.3 s for the iButtons. The mean absolute offset from the standard was 0.06 ± 0.04 °C for the eTemp Performance sensors and 0.19 ± 0.03 °C for the iButtons (*p* < 0.001). Human experiments showed no differences between eTemp Performance sensors and iButtons in mean skin temperature values during exercise in the heat. The eTemp Performance sensors are recommended for assessment of mean skin temperature determination since they have a response time in water that is 2.5 times shorter than that of the iButtons, a lower mean absolute offset from the standard than iButtons, and yield similar mean skin temperatures during exercise in the heat.

## Introduction

The human skin is the largest organ of the human body and plays an essential role in temperature regulation [1]. The skin contains cold and warm receptors that transmit information about the body’s thermal state to the central nervous system [2]. In hot conditions, cutaneous blood flow increases, enhancing heat dissipation to the environment through radiation, convection, and conduction. In addition, sweat glands produce sweat, which cools the body as it evaporates from the skin surface. In cold conditions, local skin temperature—particularly in the extremities—may decrease to near-freezing value [3]. Under heat stress, temperature differences between skin measurement sites become smaller, and mean skin temperature can reach up to 36 °C when protective clothing is worn [4].

Thus, mean skin temperature (MST) of humans is an important physiological parameter that reflects the human response to ambient conditions, and it reflects the thermal status of the human body in combination with the body core temperature. MST is a main determinant of thermal behavior [5] and can be assessed using infrared thermography [6] or temperature sensors directly applied at the skin surface [7]. Temperature sensors directly connected to the skin are generally thermocouples or thermistors. The accuracy and response time of these sensors are optimal when the sensors are thin and not ‘protected’ by a foam layer [8].

At least seven skin locations are required to obtain a good estimate of MST [9]. Several studies discuss the optimal locations and number of measurement sites [10, 11]. ISO standard 9886 defines four, eight, or 14 locations to measure the MST [12]. The measurement of MST has become much easier with the introduction of wireless measurement systems such as iButtons [13]. However, these systems do not allow for real-time read out of the MST, with the risk that erroneous data may be recorded that cannot be detected and corrected before the end of the session is reached. Recently, a new skin temperature sensor was developed that enables real-time wireless measurement.

The aim of this study was to evaluate the validity and accuracy of these so-called eTemp Performance skin temperature sensors in in- and ex-vivo circumstances. For that purpose, a water bath experiment was conducted in which temperature measurements of the new sensor were compared with the existing wireless sensor (iButton) and a calibrated gold standard thermometer. Furthermore, an in-vivo experiment was performed during exercise in the heat to evaluate differences between the new sensors and iButtons in mean skin temperature and ease-of-use.

## Methods

### Experimental setup

Two iButtons and five eTemp Performance sensors were immersed in a Tamson thermostat water bath with stirring (TLC15-5; Tamson Instruments B.V., Bleiswijk, the Netherlands). Another water bath was prepared at approximately 37 °C to determine the time response. The temperature of the water in the thermostat bath or in the 37 °C bath was measured using a Greisinger GMH 3750 (GHM Messtechnik GmbH, Regenstauf, Germany) reference. The Greisinger has a calibration certificate stating that the accuracy is better than 0.07 °C. The measurements were performed at 1 s intervals.

The iButtons (Dallas Maxim, Dallas, USA) are of type DS1922L and have a semiconductor temperature sensor, a computer chip with a real-time clock and memory, and a 3 V Lithium battery enclosed in a 16 × 6 mm^2^ stainless steel can. They have a diameter of 17.7 mm and, thickness of 6.4 mm and mass of 2.895 g. The temperature sensor is located toward the top of the iButton. Manufacturing specifications are: a temperature range between − 10 and + 65 °C, an accuracy of 0.5 °C with a precision of 0.0625 °C, and a 16-bit mode AD converter is used. The output value given by the iButton is the instantaneous value at that particular moment.

**Fig. 1:**
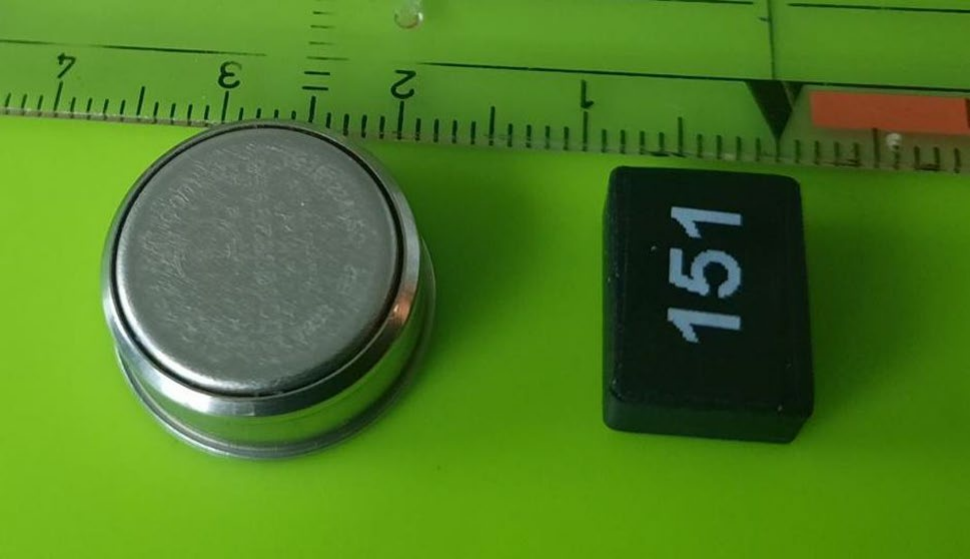
Ibutton (left) and eTemp Performance sensor (right)

The eTemp Performance sensors (Bodycap, Hérouville Saint Clair, France) are boxes sized 12.4 × 9.5 × 5.8 mm and weigh 1.322 g (Fig. 1). The outer layer is made of plastic which is thinner and conductive on the bottom side. The temperature sensor is located adjacent to the thin plastic layer. A Bluetooth system is built in that communicates with a laptop that has to be within about 10 m range. The specified temperature range is − 30 to 55 °C, the temperature accuracy is ± 0.1 °C; the temperature resolution is 0.01 °C and 3600 temperature values can be stored into the sensor’s internal memory.

### Time response of the sensors

**Fig. 2:**
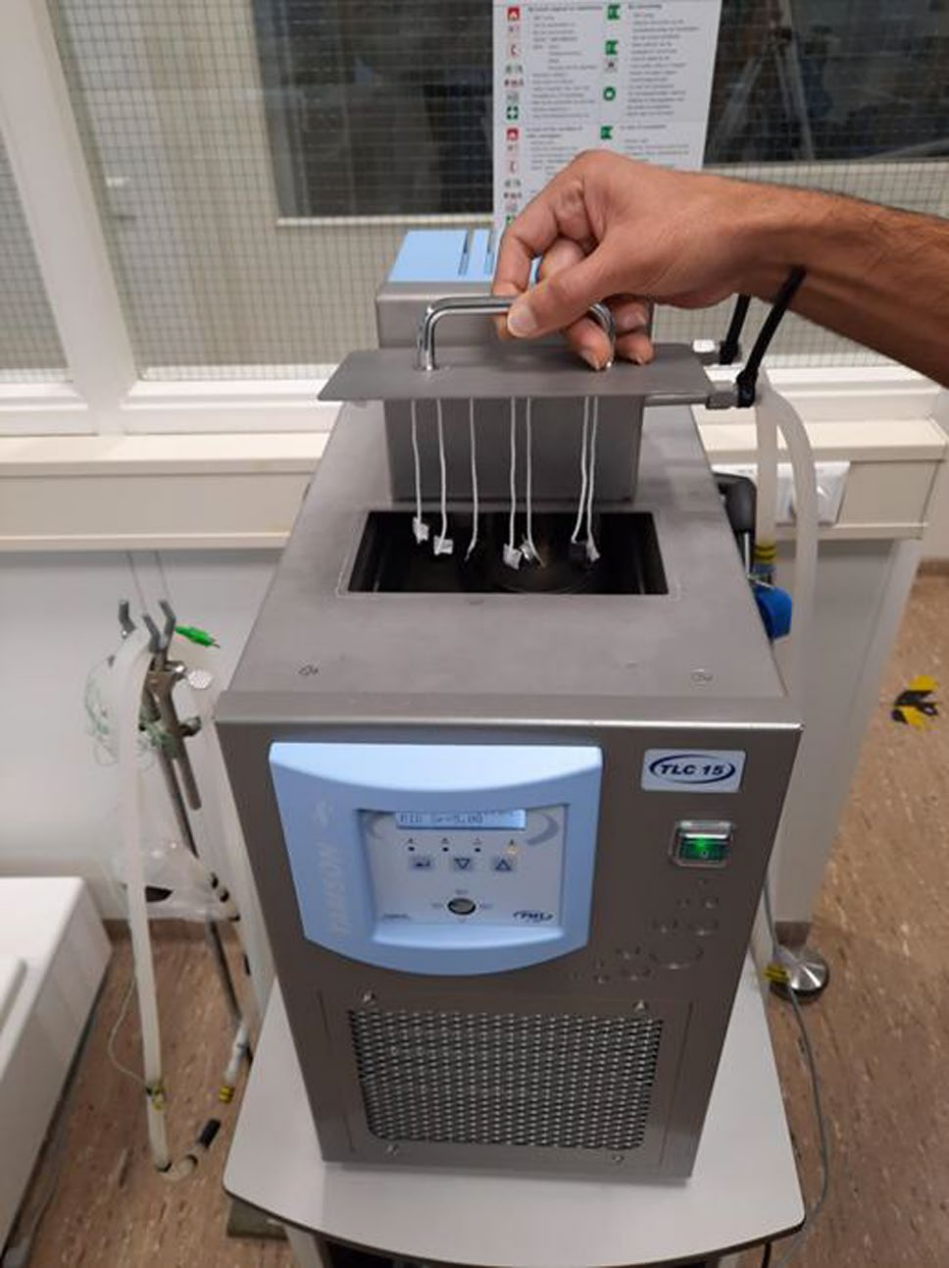
Setup of the experiment. The sensors were attached to the lid of the Tamson bath

The two iButtons and five eTemp Performance sensors were attached to the lid of the thermostat water bath so that they could hang freely in the water bath (Fig. 2). The Greisinger sensor was in the 37 °C water bath and the temperature of the thermostat bath was read from the display. The time constant was determined by quickly moving the sensor from a 5 °C water bath to a 37 °C water bath, and measuring the time until the sensor temperature was similar to the water bath temperature. The time constant, also called Tau, is defined as the amount of time it takes from starting temperature to reach 63.2% of the final steady-state value. Subsequently, the sensors were placed in the 5 °C water bath again. This procedure was performed twice. The time response was fitted with formula a * exp (b * time) + c, with time in seconds.

### Accuracy

In the experiment to determine the mean absolute offset, the temperature of the water bath was raised over the course of ~ 4 h from 10 to 40 °C in steps of 10 °C, and then lowered from 40 to 30 °C. During this time period, temperature measurements were performed using three iButtons and five eTemp Performance sensors, along with the Greisinger standard as a reference measurement. One eTemp Performance sensor was not included because it ran out of battery power immediately before returning to the 30 °C temperature setting. The temperature was maintained stable for approximately 10 min, for which a minimum of 7 min was used to calculate the mean temperature values and standard deviations. During the accuracy experiment, the temperature values measured with the eTemp Performance sensor, iButton and Greisinger sensor were continuously recorded at 10 s intervals. Differences between the gold standard and eTemp Performance/iButtons are referred to as offset or accuracy.

The software used to activate, read out, and process the data of the iButtons was OneWireViewer (https://www.iButtonlink.com/products/maxim-1-wire-viewer). The software used to activate and process the information from the eTemp Performance sensors was custom-made. Manual mode was used. Since the maximum measurement time was 1 h for 1 s registrations of five sensors to determine the response time, the setting was changed to 10 s intervals for the measurement of the accuracy. The software enables the calculation and storage of mean skin temperature based on ISO 9886.

### Human experiment

One volunteer (male, 21 years old, stature 191 cm, and body mass 69 kg) performed cycling exercise for 30 min at 120 W, followed by brief weighing outside the climatic chamber, after which the participant cycled for at least 30 min at a core temperature of 38.8 °C (controlled hyperthermia). Thereafter, the participant sat on a chair for 10 min. The climatic chamber was set at 35 °C and 65% relative humidity. IButtons and eTemp Performance sensors were located at four standard locations according to ISO 9886 [12]: neck (1), shoulder (2), hand (3), and shin (4), and a weighted average skin temperature was calculated according to the ISO guidelines. Sensors were placed adjacent to the skin, and were applied to the skin using fixomull tape (BSN Medical, Hamburg, Germany). Power of the sensors was monitored.

### Statistics

The CSV files from the iButtons and CSV data files from the eTemp Performance system were processed in Microsoft Excel and analyzed using Statistica software (TIBCO Software Inc. (2020). Data Science Workbench version 14. http://tibco.com). Differences between iButtons and eTemp Performance sensors were determined using the t test for time response and accuracy.

## Results

### Time response

**Fig. 3:**
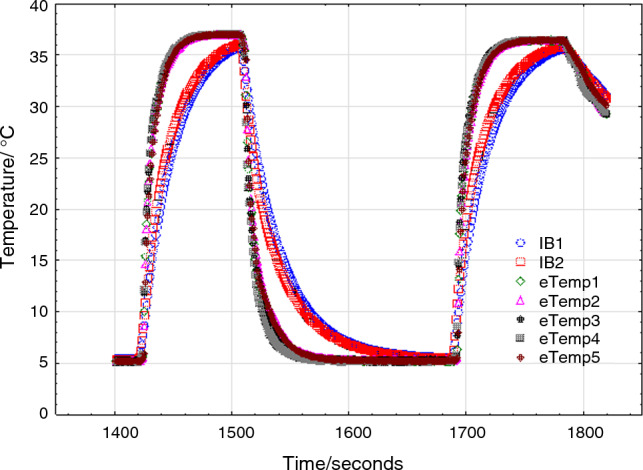
Time response of the 7 sensors in the first part of the experiment. *IB* Ibutton, *eTemp* eTemp Performance sensor

The time response curves of the sensors are shown in Fig. 3. Water temperature of the warm water bath was 37.19 °C when the sensors were immersed for the first time and dropped to 37.01 °C during the immersion as determined using the Greisinger system. Water bath temperature was 36.55 °C at the second immersion and dropped to 36.45 °C. The time constant for the eTemp Performance sensors was 10.5 ± 1.1 s and 25.8 ± 3.3 s for the iButtons based on two times a temperature increase after immersion in warm water and one time immersion in cold water (*t* test, *p* < 0.001).

The Root Mean Square Error (RMSE) of the curves fitted with formula a * exp (b * time) + c, was always better than 1 °C and averaged 0.4 °C. The b-component in the fit curve was -0.04 ± 0.01 for the iButtons and − 0.11 ± 0.01 for eTemp sensors. The b-values did not differ significantly (p > 0.05) between heating and cooling for the iButton and eTemp sensor systems.

### Accuracy

**Fig. 4:**
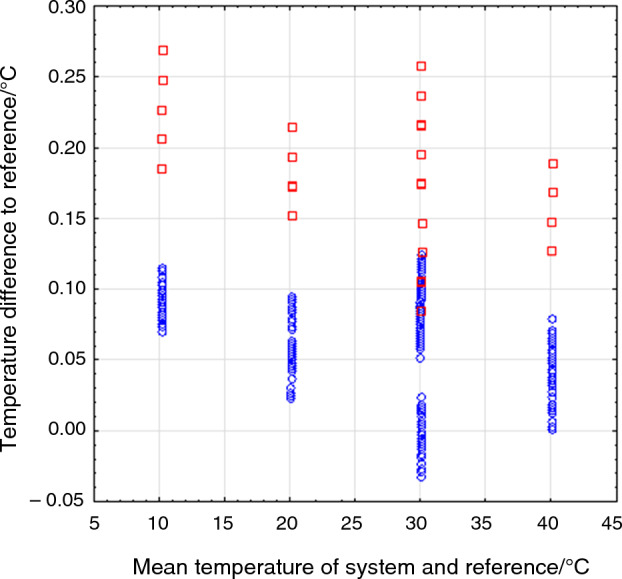
Bland–Altman plot of the iButtons (red) and eTemp Performance sensors (blue)

The results are presented in a Bland–Altman plot (Fig. 4). The mean offsets for the eTemp Performance sensors were 0.06 ± 0.04 °C and 0.19 ± 0.03 °C for the iButtons, respectively (*t* test, *p* < 0.001). The battery voltage was over 2 V at the start for all four eTemp Performance sensors, but one sensor showed a drop during the experiment to 1.2 V.

### Human experiment

**Fig. 5:**
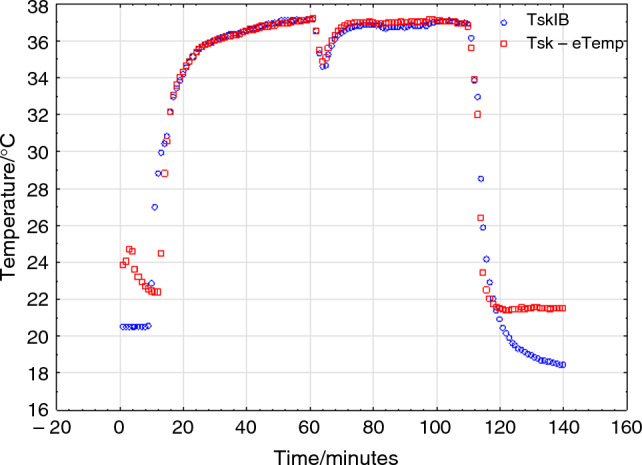
Mean skin temperature of the iButtons and eTemp Performance sensors. *TskIB* skin temperature of iButtons, *Tsk-eTemp* skin temperature of eTemp Performance sensors

**Fig. 6:**
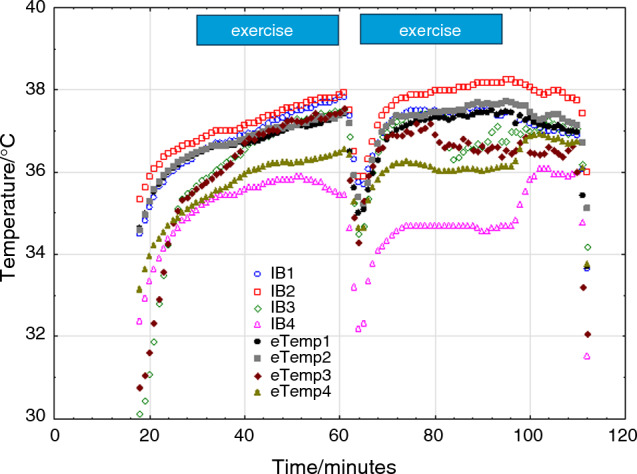
Values for each sensor. *IB* iButton, *eTemp* eTemp Performance sensors. 1 = neck; 2 = shoulder; 3 = hand; 4 = shin

Figure 5 shows the mean skin temperatures during the human experiment measured using the iButton and eTemp Performance sensors, while the values for each individual sensor/site are shown in Fig. 6.

Average skin temperature was not significantly different between the iButton and eTemp Performance sensors (Fig. 5). However, temperature values measured at the same location of the body differed considerably between both sensors (Fig. 6). For instance, at the shin skin temperature measured with the eTemp Performance sensor was > 1 °C higher compared to the iButton in the second part of the experiment, while shoulder temperature was higher when measured with the iButtons. During cycling, the temperature of the shin is relatively low as compared to other skin temperatures due to the enhanced convective and evaporative heat loss related to the movement of the lower legs. The metal iButton sensor seems to be more affected than the eTemp Performance sensors due to the higher thermal conductance of the IButton sensors.

Voltage of all eTemp Performance sensors dropped gradually from 2.5 to 1.8 V over the course of 140 min.

## Discussion

The aim of this study was to evaluate the validity and accuracy of the eTemp Performance skin temperature sensors in in- and ex-vivo circumstances. We found that the eTemp Performance sensors had a response time of 10.5 ± 1.1 s, which is much faster than the response time of iButtons (25.8 ± 3.3 s). This difference is likely related to the mass / thermal inertia of the sensor systems. The mass of the iButtons is more than two times that of the eTemp Performance sensors. Thus, for experiments in which fast skin temperature changes can be expected and the skin temperature response is an outcome variable, such as moving from cold to warm rooms or vice versa, the eTemp Performance sensors are a better option than iButtons. Moreover, we found that the eTemp Performance sensors deviated less from values of the Greisinger reference and thus were more accurate on average than the iButtons (0.06 ± 0.04 °C versus 0.19 ± 0.03 °C, respectively).

There can be substantial differences between sensors. One iButton had an absolute offset of approximately 0.27 °C and one eTemp Performance sensor had an offset of 0.13 °C (Fig. 4). These deviations in some sensors can be masked using multiple sensors on the skin, as is common for mean skin determination. For determination of mean skin temperature, the time response benefits of the eTemp Performance sensors are likely more important than the accuracy benefits, since mean skin temperature is a weighted average of up to 14 skin locations and variations in placement may cause larger errors than the absolute difference between the two systems. When the sensors on the legs, for instance, are relocated slightly more distally, this may result in considerably lower temperatures [1]. In the human experiment we also observed considerable variation between sensors (Fig. 6). It is not unlikely that this variation is related to local differences in skin blood perfusion, and may also be due to the pressure applied to the skin by the fixomull tape. Still, the measurements showed to be comparable for mean skin temperature between eTemp Performance and iButton sensors, illustrating the need to use multiple sensors to determine mean skin temperature reliably.

Both sensors are wireless and, therefore, convenient for human research. We experienced that the eTemp Performance sensors were easy to use due to their small size and clear numbers on the front of the sensors. The iButtons can be numbered using a permanent marker on the back and are also easy to handle. A considerable advantage of the eTemp Performance system is that it offers a live read out of temperature data. IButtons do not have live read-out options. Using eTemp Performance, live temperature data can be displayed on a laptop, but the range of ~ 10 m for a proper connection limits its current monitoring possibilities in field settings. iButtons are widely used in scientific research and are known for their robustness and reliability with regard to data collection (i.e., limited missing data). Using eTemp Performance, we lost a small part of the data owing to connection problems, which must be improved. Both sensor systems offer great advantages over traditional wired thermocouple and thermistor systems in ease of use since wires are not necessary. However, for very fast changes in skin temperature wired systems still are the preferred configuration. In that case it is important that the tape to fix the sensor to the skin is as small as possible or even absent [14]. Infrared imaging of the human skin seems a non-invasive and practical way to measure skin temperature, but it can only measure body parts uncovered by clothing and is affected by environmental, individual and technical factors making this method error prone [15].

Both sensors were small; however, their outer materials differed. IButtons have a metal outer layer and the eTemp Performance has a plastic outer layer. In cold environments the metal outer layer may enhance the risk for local cold injuries.

Another difference between the two sensors is the battery. Where iButtons have an internal battery that cannot be replaced, the eTemp Performance sensors have to be charged. Both are feasible in laboratory and field settings. Charging sensors are preferred in terms of sustainability. In addition, both sensors can store approximately the same number of datapoints (3000 for iButtons and 3600 for eTemp Performance). The eTemp Performance sensors have reloadable batteries that empty quicker than fixed batteries in the iButton system. Battery power dropped from 2.4 to 1.8 V over 140 min and were still operational then. For experiments lasting more than about 4 h, replacing the sensors with fully loaded new sensors has to be considered. Finally, specific types of iButtons allow for measuring humidity where the eTemp Performance sensors currently do not. Adding humidity can be beneficial, depending on the specific research question.

Some limitations of the current study need to be considered. First, the experience with human experiments is still limited and future experiments can provide a clear image of the durability of the eTemp Performance system and battery power maintenance. Second, the time response and accuracy of the systems were evaluated in water and not in air. Water conducts heat much better than air and is therefore more suited to determine fast time responses. It is expected that the observations in water extend to air, but it is encouraged to evaluate this in future studies. Third, the eTemp Performance system that we evaluated was new, and possible changes in time constant and accuracy over time due to aging were not evaluated. Finally, the number of eTemp Performance sensors and iButtons used in the study varied between experiments from 2 to 5 for practical reasons; an equal number of sensors of each system might have led to a more fair comparison.

In conclusion, this study showed that eTemp Performance sensors outperform iButtons regarding time response (10.5 ± 1.1 versus 25.8 ± 3.3 s) and accuracy (0.06 ± 0.04 °C versus 0.19 ± 0.03 °C), are easy to use and provide similar values in human experiments to determine mean skin temperature.

## Data Availability

Research data supporting this publication are freely available at: [10.6084/m9.figshare.28375601.v1] (https:/doi.org/10.6084/m9.figshare.28375601.v1) for the metafile; [10.6084/m9.figshare.28375607.v1] (https:/doi.org/10.6084/m9.figshare.28375607.v1) for the datafile with time response; [10.6084/m9.figshare.28375604.v1] (https:/doi.org/10.6084/m9.figshare.28375604.v1) for the datafile with accuracy; [10.6084/m9.figshare.28375610.v1] (https:/doi.org/10.6084/m9.figshare.28375610.v1) for the datafile with human data.
